# *Candida auris* surveillance in the Military Health System: a multidrug-resistant threat

**DOI:** 10.1017/ash.2025.10204

**Published:** 2025-11-11

**Authors:** J. Joseph Brough, Graham C. Ellis, Sara L. Robinson, John L. Kiley, David D. Blaney, Michelle Dressner, Jason Stam, Jamie Myers, Ethan Green, Michelle Wagner, Kelly Andrews, Daniel Krauth, Dianne Frankel

**Affiliations:** 1 Walter Reed National Military Medical Center, Bethesda, MD, USA; 2 Naval Health Research Center San Diego, San Diego, CA, USA; 3 Multidrug-Resistant Organism Repository and Surveillance Network, Silver Spring, MD, USA; 4 Brooke Army Medical Center, San Antonio, TX, USA; 5 Office of the Command Surgeon, HQ U.S. Africa Command, Stuttgart, Germany; 6 Centers for Disease Control and Prevention, Atlanta, GA, USA; 7 Naval Medical Center San Diego, San Diego, CA, USA

## Abstract

**Objective:**

To evaluate the potential sources and current screening strategies for the multidrug-resistant fungal pathogen *Candida auris* in the US Military Health System (MHS).

**Methods:**

Utilizing the Multidrug-Resistant Organism Repository and Surveillance Network (MRSN), 6 instances of *C. auris* colonization or infection were identified within the MHS in 2024. Relevant medical and social history, drug susceptibilities, and next-generation genetic sequencing were obtained from MRSN and the electronic medical record. Hospital screening protocols for *C. auris* were reviewed in the affected facilities.

**Results:**

One case of *C. auris* infection and 5 cases of *C. auris* colonization in 2024 were identified in the MHS. Only 1 case of colonization was likely related to international travel; 5 patients had no recent travel history before infection or colonization. One patient was an active duty service member. Prior hospitalizations and infections were the most common risk factors present in each case. Two isolates had antimicrobial susceptibilities analyzed, both of which suggested resistance to fluconazole. Two of the 3 facilities had *C. auris* screening protocols in place to screen select individuals with risk factors; however, only 1 of the 6 cases presented was identified through these screening protocols. No cases of nosocomial transmission were found.

**Conclusions:**

*C. auris* remains a formidable threat to the MHS, with 6 cases identified in 3 treatment facilities, with 2 isolates demonstrating resistance to azoles. Screening protocols should reflect the domestic and international threats of this pathogen.

## Introduction

*Candida auris* (renamed to *Candidozyma auris* in 2024, however, will be referred to as *Candida auris* for the purpose of clarity in this paper)^1^ is an emerging multidrug-resistant (MDR) fungal pathogen that poses a nosocomial threat to immunosuppressed and otherwise susceptible patients and potentially impacts health systems worldwide. Since first reported as a cause of human disease in Japan and South Korea in 2002,^2^ and subsequently in the United States in 2016, global cases have risen precipitously.^2–4^ Bloodstream infections have been associated with an all-cause mortality of 30%–72%,^2–4^ with 40%–86%^2,3^ of isolates harboring azole resistance. Total number of cases in the United States rose from 53 to 4,514 between 2016 and 2023.^5^ The Centers for Disease Control and Prevention (CDC) Antimicrobial Resistance Laboratory Network reported a 7% increase in azole resistance among *C. auris* isolates from 2019 to 2020, restricting treatment options.^1^ *C. auris* has also been shown to contaminate the hospital environment even after room disinfection, heightening the risk of nosocomial outbreaks.^6^ As such, CDC classified *C. auris* as an “urgent threat” to public health in the 2019 and 2021–2022 *Antibiotic Resistance Threats in the United States* reports.^7^

Reducing the risk of *C. auris* nosocomial transmission requires identifying and isolating colonized patients early. Hospital outbreaks have been reported internationally and are frequently attributed to deficient isolation and hygiene practices.^3^ Although decolonization with antimicrobials is not currently recommended, CDC advocates for the implementation of isolation and infection control procedures in cases of *C. auris* colonization. The US Military Health System (MHS) cares for all active duty US service members, retirees, and their dependent family members. Given the global breadth of the US military, the MHS sees patients across the world and has a particular interest in preventing nosocomial transmission of emerging infectious diseases. Additionally, the military has a history of being particularly exposed and susceptible to MDR pathogens and could also be a vehicle to spread MDR organisms (MDROs) to the US population if precautions are not taken.^8^ Here, we present 1 case of *C. auris* infection and 5 cases of *C. auris* colonization in MHS patients in 2024 identified by the Department of Defense Multidrug-Resistant Organism Repository and Surveillance Network (MRSN). Three hospitals in the MHS are included in this study, hereafter referred to as Hospital A, Hospital B, and Hospital C. Table 1 provides a summary of the cases. This is, to our knowledge, the first known case series of *C. auris* involving a healthcare network with presence in multiple nations that also investigates potential sources and screening protocols for the pathogen.

**Table 1.** Summary of *Candida auris* cases in the Military Health System, 2024

Case and location	Age/sex	Active duty (Y/N)	Colonization or disease?	International travel history	Recent prior residency	Recent prior hospitalizations?	History of MDRO infections?	Clade of isolate
Case 1: Hospital A	38 y.o. male	No	Disease	No	Skilled nursing facility	3 in year prior	ESBL endocarditis; CRAB bacteremia	III (African)
Case 2: Hospital A	74 y.o. female	No	Colonization	No	Home	1 in past year	No	III (African)
Case 3: Hospital B	71 y.o. male	No	Colonization	No	Home	1 in past year	No	I (South Asian)
Case 4: Hospital B	24 y.o. male	Yes	Colonization	None (no deployments)	Military barracks	No	No	N/A
Case 5: Hospital B	74 y.o. male	No	Colonization	No	Home, but had extended stay in inpatient rehab in prior 2 months	4 in year prior, including stay at inpatient rehab	*Candida albicans* pyelonephritis	N/A
Case 6: Hospital C	31 y.o. Male	No	Colonization	Kenya (hospitalized); Germany (hospitalized)	Home, but traveling internationally at time of injury	No	No	III (African)

Note. MHS, Military Health System; y.o., years old; MDRO, multidrug-resistant organism; ESBL, extended-spectrum beta-lactamase; CRAB, carbapenem-resistant *Acinetobacter baumannii*; N/A, not available.

## Case 1: Hospital A

A 38-year-old male with a history of advanced multiple sclerosis with contractures complicated by paraplegia, neurogenic bowel and bladder with chronic suprapubic catheter, gastrostomy tube, colostomy, multiple ischial and sacral pressure ulcers stages 2–3, recurrent urinary tract infections (UTIs), nephrolithiasis, and bilateral nephrostomy tube placement was readmitted to Hospital A for planned lithotripsy. He notably had no recent international travel history but was frequently admitted to skilled nursing facilities in the local area.

Four months before this admission, the patient was diagnosed with sacral osteomyelitis and treated with doxycycline and levofloxacin. He also had a *Candida albicans* UTI treated with fluconazole followed by fluconazole suppressive therapy for 2 months. Two months prior to admission, he was seen at an outside hospital initially for bilateral lower extremity tenotomies and contracture releases. However, his course was complicated by vancomycin-resistant *Enterococcus* endocarditis and carbapenem-resistant *Acinetobacter baumannii* bacteremia, and he was started on appropriate antimicrobials. Prior to lithotripsy, a urine culture was obtained, and *C. auris* was isolated. Amphotericin B was started and then stopped after he developed a significant infusion reaction. Lithotripsy was rescheduled for after completion of the antibacterial course for his endocarditis.

Shortly after his planned lithotripsy, the patient became tachycardic, febrile, and hypotensive. At this time, *C. auris* was suspected as a potential causative organism for his decompensation given prior isolation in the urine, and the patient was started on micafungin in addition to daptomycin and ceftazidime/avibactam (amphotericin B was initially avoided given prior reaction). However, the patient continued to clinically worsen, and blood cultures collected 2 days later grew isolates of *C. auris*, prompting initiation of amphotericin B with aggressive pretreatment with steroids and antihistamines for 2 weeks. The patient completed this therapy and was discharged from the hospital.

**Figure 1.**
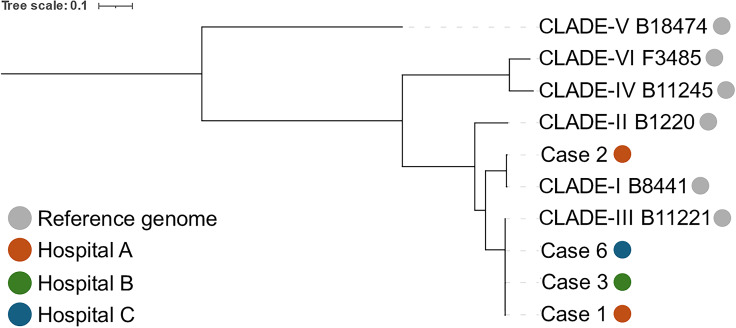
Genetic relatedness of *C. auris* isolates from the Military Health System (MHS), 2024. Core genome phylogeny for 4 *C. auris* isolates from the MHS (Cases 1, 2, 3, and 6) and 6 reference isolates from GenBank. The phylogenetic tree was built using the GTR-GAMMA model in RAxML v8.2.11 and a core genome alignment from Panseq (fragmentation size of 500 base pairs to find sequences with ≥95% identity in ≥95% of the isolates, which resulted in a 13-Mb core genome and an alignment of 306,480 informative sites). All isolates except 1 isolate from Hospital A were found to be clade III (African); 1 isolate from Hospital A was found to be clade I (South Asian). Cases 4 and 5 were isolates that were unavailable for sequencing.

Molecular analysis with next-generation genetic sequencing (NGS) was performed at MRSN in Silver Spring, Maryland, and compared with other *C. auris* isolates collected from the MHS (Figure 1). The isolate was identified as clade III (African). Antimicrobial susceptibility testing revealed a minimum inhibitory concentration (MIC) to fluconazole of >64 µg/mL (Table 2). Environmental investigation was performed at Hospital A through swabs of the sink and surfaces in the patient’s room after isocharge. No residual colonies of *C. auris* were isolated from those swabs.

**Table 2.** Susceptibility testing of Case 1 *Candida auris* isolate

Antibiotic	MIC (µg/mL)	CDC-suggested MIC breakpoint^a^	Interpretation^b^
5-flucytosine	.25	N/A^c^	N/A
Amphotericin B	.5	≥2	Susceptible
Micafungin	.25	≥4	Susceptible
Fluconazole	>64	≥32	Resistant
Posaconazole	<.03	Use fluconazole as a surrogate	Resistant

Note. MIC, minimum inhibitory concentration; CDC, Centers for Disease Control and Prevention; N/A, not applicable.

a Per CDC^14^: “There are currently no established *C. auris*-specific susceptibility breakpoints. Therefore, breakpoints are defined based on those established for closely related *Candida* species and on expert opinion. Correlation between microbiologic breakpoints and clinical outcomes is not known at this time. Therefore, the information…should be considered as a general guide and not as definitive breakpoints for resistance.”

b Based on CDC-suggested breakpoints.^14^

c CDC does not recommend breakpoint for 5-flucytosine.

## Case 2: Hospital A

A 74-year-old female with a history of heart failure, diabetes mellitus, chronic obstructive pulmonary disease, and hypothyroidism was admitted to the intensive care unit of Hospital A for severe abdominal pain and distention, acute respiratory failure, and hypotension. She had no significant infectious history, and she lived in an apartment independently with no recent international travel history, but she had frequent admissions to outside hospitals and skilled nursing facilities for heart failure exacerbations. She was started on vancomycin and piperacillin/tazobactam for sepsis with a potential gastrointestinal, pulmonary, or urinary source. After initial improvement, she subsequently experienced symptomatic hypotension requiring vasopressors thought to be related to either an infectious or cardiogenic etiology. *C. auris* was isolated in 3 separate urine cultures that were obtained as part of a broad sepsis workup but ultimately was not treated given the lack of urinary-specific symptoms. Throughout her hospital course, she never developed symptoms of a UTI, and *C. auris* was not isolated elsewhere. She developed Ogilvie’s syndrome during her hospital course and ultimately died after spontaneous bowel perforation. Molecular analysis with NGS was performed at MRSN (Figure 1); the isolate was identified as clade III (African).

## Case 3: Hospital B

A 71-year-old male with a history of multiple transient ischemic attacks (2011, 2024), prostate cancer and prostatectomy in 2016, chronic obstructive pulmonary disease, urinary retention with chronic indwelling Foley catheter, and recent admission for pneumonia requiring intubation presented to the emergency department with nausea and vomiting. He lived in the community and did not report any recent international travel. He also complained of pain at the tip of his penis associated with his Foley catheter. Urinalysis revealed pyuria, leukocyte esterase, and budding yeast. Although fluconazole was started initially, the urine cultures ultimately grew *C. auris.* Infectious disease specialists were consulted, and they recommended exchanging the urinary catheter and stopping systemic antifungal therapy given there were no systemic signs of infection. He recovered from his episode of nausea and vomiting with supportive care and was eventually discharged without complications. Molecular analysis with NGS was performed at MRSN (Figure 1); the isolate was identified as clade I (South Asian).

## Case 4: Hospital B

A 24-year-old active duty male without any significant past medical or international travel history (to include military deployments) was participating in military training exercises within the United States and was admitted from the field for shortness of breath, cough, fevers, chills, and night sweats for 2 weeks. After admission, he decompensated into septic shock from pneumonia. While receiving ventilatory and pressure support, he developed cardiac arrest from ventricular tachycardia. Lower respiratory PCR was positive for *Streptococcus pneumoniae*. He eventually recovered and completed a 7-day course of meropenem and linezolid. After discharge, urine cultures obtained during his hospitalization as part of a broad infectious workup for septic shock were positive for *C. auris*; however, this was not treated given the patient did not exhibit urinary symptoms and had fully recovered after receiving appropriate treatment for community-acquired pneumonia and sepsis. Due to a lack of access to this isolate, it was not sequenced, and the clade of the isolate was not found.

## Case 5: Hospital B

A 74-year-old male with a history of heart failure, atrial fibrillation, type 2 diabetes, a fall (2023) resulting in central cord syndrome, and neurogenic bladder further complicated by *C. albicans* pyelonephritis and *Clostridioides difficile* infection presented to the emergency department with weakness and poor appetite. The patient was living in the community and had no recent international travel history. He was experiencing urinary leakage for several months and underwent urodynamic testing 1 week prior to admission which revealed poor bladder capacity and a contractility index consistent with urinary retention. Acute kidney injury was diagnosed, thought to be secondary to both poor oral intake and chronic urinary retention, and he was admitted to the hospital for intravenous (IV) fluid support and urology evaluation. Acute kidney injury improved after administration of IV fluids. A urine culture was performed and initially grew *Candida* not identified to the species level. The patient received fluconazole for 3 days, but it was discontinued given the absence of urinary symptoms. Infectious disease specialists were consulted and did not recommend further antimicrobial therapy given the absence of symptoms and presumed colonization. The patient was discharged after resolution of acute kidney injury, with outpatient urology follow-up for management of neurogenic bladder. Five days after discharge, *C. auris* was identified from the urine culture. Infectious disease specialists were re-consulted and recommended no treatment of *C. auris*, given the absence of symptoms. Due to lack of access to this isolate, it was not sequenced and the clade of the isolate was not found.

## Case 6: Kenya, Germany, and Hospital C

A 31-year-old male MHS beneficiary with no significant past medical history suffered polytrauma during a skydiving accident outside of Mombasa, Kenya. The patient was admitted to the intensive care unit at Mombasa Hospital for initial stabilization and surgical management. During hospitalization, he received a blood transfusion, had suprapubic and subclavian central venous catheters placed, and underwent external fixation of right tibial/fibular and right hemipelvis fractures. After 2 days, he received additional blood transfusions in transit to a hospital in Germany, where he had orthopedic fixation adjusted. After 2 days in Germany, he was transferred to Hospital C in the United States for definitive management/rehabilitation. Prior to this injury, he had minimal exposure to healthcare settings.

Per MEDEVAC isolation protocol, the patient was placed in contact isolation upon arrival to Germany, which was continued upon transfer to Hospital C. Perirectal surveillance swabs for MDROs were performed per protocol, identifying extended-spectrum beta-lactamase (ESBL)-producing *Escherichia coli* and ESBL-producing *Klebsiella pneumonia*. Per protocol, upon arrival at Hospital C, groin/axillary swabs for *C. auris* were collected and found to be positive for *C. auris* colonization.

The patient displayed no clinical evidence of fungal infection (systemic or localized); therefore, no antifungal treatment was initiated. Isolation precautions, instituted on admission to the MHS, were continued throughout his admission, the course of which was unremarkable from an infectious disease standpoint. No nosocomial transmission of *C. auris* was detected. Through molecular analysis at MRSN, the isolate was identified as clade III (African) and found to carry the VF125AL mutation in ERG11, indicating resistance to fluconazole. Antifungal susceptibility testing was performed at MRSN, resulting in a MIC of 256 µg/mL to fluconazole (Table 3).

**Table 3.** Susceptibility testing of Case 6 *Candida auris* isolate

Antibiotic	MIC (µg/mL)	CDC-suggested MIC breakpoint^a^	Interpretation^b^
5-flucytosine	.12	N/A^c^	N/A
Amphotericin B	1	≥2	Susceptible
Anidulafungin	.25	≥4	Susceptible
Caspofungin	.25	≥2	Susceptible
Micafungin	.25	≥4	Susceptible
Fluconazole	>256	≥32	Resistant
Itraconazole	.25	Use fluconazole as a surrogate	
Posaconazole	.12		Resistant
Voriconazole	.25		Resistant

Note. MIC, minimum inhibitory concentration; CDC, Centers for Disease Control and Prevention; N/A, not applicable.

a Per CDC^14^: “There are currently no established *C. auris*-specific susceptibility breakpoints. Therefore, breakpoints are defined based on those established for closely related *Candida* species and on expert opinion. Correlation between microbiologic breakpoints and clinical outcomes is not known at this time. Therefore, the information…should be considered as a general guide and not as definitive breakpoints for resistance.”

b Based on CDC-suggested breakpoints.^14^

c CDC does not recommend breakpoint for 5-flucytosine.

## Discussion

CDC currently recommends screening for *C. auris* in patients with known exposure to the pathogen, patients with exposure to high-acuity postacute care facilities, including long-term acute care hospitals and ventilator-capable skilled nursing facilities, and patients who have had an overnight stay in a healthcare facility outside the continental United States (OCONUS). CDC also recommends considering screening individuals at higher risk for infection, including individuals on ventilatory support, infection with other MDROs, or with multiple IV lines or medical devices.^9^ Our case series highlights the need for screening patients with exposure to OCONUS healthcare facilities (Case 6), as well as individuals with recurrent hospital stays, a history of infections or colonization with MDROs (Cases 1, 2, 3, and 5), or ventilatory support (Case 4).

Molecular analysis also provides some insight into the origin of the *C. auris* isolates. As illustrated in Figure 1, 3 of the 4 genotyped isolates were classified as clade III (African), with Case 4 classified as clade I (South Asian). Clade I isolates have been identified predominantly in the northeast United States, while clade III isolates have been identified in Indiana, Texas, Florida, and California. A 2019 study revealed a predominantly clade III outbreak in Los Angeles, California.^10^ Although the exact location of transmission cannot be determined through genetic analysis, the genetic isolates from this series support the domestic presence of *C. auris* with multiple geographic and genotypic origins and are consistent with the genetic makeup of *C. auris* that has been transmitted domestically in the past.^11^ The identification of a Clade III isolate in Case 6 supports that transmission likely occurred in Africa as opposed to Germany, as Clade III has historically been more frequently isolated in Africa than Europe.^12^

The MHS routinely manages cases of illness and injury sustained outside the continental United States (OCONUS) from the point of injury to definitive care in the United States. In both peacetime and wartime, patient evacuations are received in military treatment facilities (MTFs) spanning the spectrum of care, from field-based or local national facilities OCONUS to more definitive care hospitals. Large-scale combat operations such as the conflicts in the Middle East saw extensive patient evacuation of military service members with MDRO colonization and infection, complicating treatment and posing a nosocomial threat.^13^ In 2009, MRSN was established at the Walter Reed Army Institute of Research in response to this threat and currently performs active surveillance, antimicrobial testing, and archival analysis of MDR isolates from across the MHS and partner healthcare systems. Although not observed in our series, the potential for nosocomial spread and emerging international incidence of *C. auris* in regions where the US military is actively engaged, and the potential to cause treatment-resistant disease among MHS patients, has prompted increased interest in surveillance for this pathogen. This is especially relevant as conflicts transition to new geographic areas and medical evacuation transfer routes change.

The few MTFs that routinely receive OCONUS patient transfers for definitive or en route care institute some form of MDRO surveillance of OCONUS patient transfers, typically with skin and rectal bacterial swabs for MDR bacteria, and since 2021, some of these protocols have included *C. auris* fungal swabs.

Since 2021, Hospital A has expanded screening for MDRO and *C. auris*, per CDC recommendations, to all patients transferred to their hospital from any outside hospital, long-term care facility, or OCONUS. Lacking a robust clinical decision support system (CDSS) in the MHS electronic medical record (EMR), identification of these cases requires vigilance on the part of Infection Prevention and Control (IPAC) teams and clinical providers. In 2022, Hospital C expanded surveillance for *C. auris* in their facility by screening all patients admitted to the medical intensive care unit (MICU), regardless of admission pathway (including intra-hospital transfers) due to a lack of certain infection-control-related assets. Although still reliant upon clinical providers to ensure screening is performed, unit-based screening was trialed as an approach to optimize adherence to surveillance protocols. Hospital C collects *C. auris* axillary and groin swab specimens and utilizes ChromAgar techniques for screening. The specimen typically has a finalized result within 7 days.

At Hospital C, in the first 12 months of MICU-based surveillance, 215 groin and axillary samples were collected and processed with 1 positive sample (Case 6). Criteria-based surveillance based on CDC-recommended criteria for screening at Hospital A yielded 205 swabs in the first 8 months of 2024, none of which were positive. However, Hospital A reported the 2 cases of *C. auris* previously noted, found in urine and blood cultures drawn for diagnostic purposes. Currently, Hospital B is not performing surveillance for *C. auris,* and the cases described herein from that facility were found through routine urine cultures drawn for diagnostic purposes. Although the potential for a hospital outbreak is apparent,^3^ nosocomial transmission was not associated with any of the cases reported in this series.

Challenges of performing surveillance for this emerging pathogen include the inability of surveillance to distinguish between colonization and infection and the difficulty of adherence to surveillance protocols. First, the value of surveillance may be unclear as *C. auris* is often deemed to colonize rather than cause disease among patients from whom it is isolated. Second, in reviewing surveillance protocols across the MHS, challenges of adherence to IPAC testing protocols and collection of accurate data are apparent, especially without a CDSS. These gaps prompted the expansion of surveillance at Hospitals A and C. Despite these challenges, and although *C. auris* nosocomial transmission has not yet been reported in the MHS, our description of *C. auris* associated with critically ill patients in the MHS regardless of travel or exposure history suggests the potential for nosocomial spread. Because of this, it is imperative to implement sustained and intentional surveillance at most, if not all, MTFs that may receive high-risk patients if we are to avoid outbreaks seen in other healthcare systems.

In this article, we presented 1 case of *C. auris* clinical infection and 5 cases of colonization. One case, likely acquired in a Kenyan hospital, was identified on protocolized surveillance in an MTF upon transfer to the United States. The 5 other cases were likely attributable to domestic spread of *C. auris* in regional healthcare systems and a history of MDRO colonization; however, 3 cases were not associated with significant risk factors, and none of these cases were associated with evidence of nosocomial transmission that has been observed elsewhere. Clinical risk factor and unit-based surveillance continue across the MHS but are hindered by a lack of a CDSS integrated into the EMR. Highly effective and consistent local surveillance will be critical in identifying *C. auris* and mitigating outbreaks of MDROs more broadly within the MHS should an influx of OCONUS medical evacuations occur.

## Disclaimer

I am a military service member or employee of the US Government. This work was prepared as part of my official duties. Title 17, U.S.C. §105 provides that copyright protection under this title is not available for any work of the US Government. Title 17, U.S.C. §101 defines a US Government work as work prepared by a military service member or employee of the US Government as part of that person’s official duties. The views expressed in this article are those of the authors and do not necessarily reflect the official policy or position of the Department of the Navy, Department of Defense, or the US Government.
